# Impact of Data Editing Methods on Estimates of Smoking Prevalence, Global Youth Tobacco Survey, 2007–2009

**DOI:** 10.5888/pcd10.120202

**Published:** 2013-03-21

**Authors:** Eugene Lam, Italia Rolle, Mikyong Shin, Kyung Ah Lee

**Affiliations:** Author Affiliations: Italia Rolle, Mikyong Shin, Global Tobacco Control Branch, Office on Smoking and Health, National Center for Chronic Disease Prevention and Health Promotion, Centers for Disease Control and Prevention, Atlanta, Georgia; Kyung Ah Lee, Northrup Grumman Information Systems, Atlanta, Georgia.

## Abstract

Accuracy of self-reported data may be improved by data editing, a mechanism to produce accurate information by excluding inconsistent data based on a set number of predetermined decision rules. We compared data editing methods in the Global Youth Tobacco Survey (GYTS) with other editing approaches and evaluated the effects of these on smoking prevalence estimates. We evaluated 5 approaches for handling inconsistent responses to questions regarding cigarette use: GYTS, do-nothing, gatekeeper, global, and preponderance. Compared with GYTS data edits, the do-nothing and gatekeeper approaches produced similar estimates, whereas the global approach resulted in lower estimates and the preponderance approach, higher estimates. Implications for researchers using GYTS include recognition of the survey’s data editing methods and documentation in their study methods to ensure cross-study comparability.

## Objective

Accurate monitoring of cigarette smoking status among youth is important in addressing the tobacco use epidemic globally (1). However, the accuracy of self-reported health-risk behaviors in questionnaires may be compromised because of difficulties in recall, social desirability, and sensitivity of the question itself (2). Data editing is a mechanism to produce accurate information by excluding inconsistent data based on a set number of predetermined decision rules. Research suggests that editing procedures have potential effects on point estimates and cross-study comparability (3–5). This exploratory study compares the data editing method used in the Global Youth Tobacco Survey (GYTS) with other data editing approaches and evaluates the effect of these on estimates of smoking prevalence in GYTS to inform collaborators globally.

## Methods

GYTS, a self-administered school-based survey, uses a 2-stage cluster sample design that is grade-based and produces representative samples of students with ages ranging from 10 to 17 years. A subset of students aged 13 to 15 years is used for comparing the data within and across Word Health Organization (WHO) regions. In countries, such as small islands, where all students in the selected grades were surveyed, a census rather than a 2-stage cluster sample is conducted. The survey methods are described in detail elsewhere (6,7).

Eligible countries were selected on the basis of the following inclusion criteria: a nationally representative sample, recent completion of GYTS (2007–2009), large sample size (≥3,000 participants), and GYTS data publicly released. Of 35 eligible countries that met the inclusion criteria, 1 country from each WHO region was randomly selected for this study. Data analysis was performed on a subset of participants aged 13 to 15 years (n) among all ages in the grades selected for the survey (N). The selected countries and the year GYTS was conducted (values for n and N) are as follows: Ghana, 2009 (n/N = 4,171/8,295); Guatemala, 2008 (n/N = 3,838/5,565); Saudi Arabia, 2007 (n/N = 2,574/3,829); the Philippines, 2007 (n/N = 3,278/5,919); Slovakia, 2007 (n/N = 4,176/4,696); and Thailand, 2009 (n/N = 7,649/9,963).

Some questions from the GYTS presented the opportunity for participants to contradict themselves when responding (Table 1). Self-reported cigarette smoking on 1 or more of the past 30 days was used to determine cigarette smoking status. For this series of questions, 5 approaches were taken for handling inconsistent responses to questions regarding cigarette use: GYTS, do-nothing, gatekeeper, global, and preponderance (Table 1).

**Table 1:** Selected Global Youth Tobacco Survey (GYTS) Questions and Data Edit Approaches

Survey Question	Response Options
1. Have you ever tried or experimented with cigarette smoking, even 1 or 2 puffs?	a) Yes; b) no
2. How old were you when you first tried a cigarette?	a) I have never smoked cigarettes; b) 7 years old or younger; c) 8 or 9 years old; d) 10 or 11 years old; e) 12 or 13 years old; f) 14 or 15 years old; g) 16 years old or older
3. During the past 30 days, how many days did you smoke cigarettes?	a) 0 days; b) 1 or 2 days; c) 3 to 5 days; d) 6 to 9 days; e) 10 to 19 days; f) 20 to 29 days; g) All 30 days
4. During the past 30 days, on the day(s) you smoked, how many cigarettes did you usually smoke?	a) I did not smoke cigarettes during the past 30 days (1 month); b) Less than 1 cigarette per day; c) 1 cigarette per day; d) 2 to 5 cigarettes per day; e) 6 to 10 cigarettes per day; f) 11 to 20 cigarettes per day; g) More than 20 cigarettes per day
5. During the past 30 days, how did you usually get your own cigarettes?	a) I did not smoke cigarettes during the past 30 days (1 month); b) I bought them in a store, shop, or from a street vendor; c) I bought them from a vending machine; d) I gave someone else money to buy them for me; e) I borrowed them from someone else; f) I stole them; g) An older person gave them to me; h) I got them some other way
6. During the past 30 days, did anyone refuse to sell you cigarettes because of your age?	a) I did not try to buy cigarettes during the past 30 days (one month); b) Yes, someone refused to sell me cigarettes because of my age; c) No, my age did not keep me from buying cigarettes

**Data Edit Approach**	**Description**

GYTS	Logic checks for age in question 2 and logic checks for smoking status between questions 1 and 2, 1 and 3, 3 and 4. Inconsistent responses were considered missing.
Do-nothing	Response to each question was taken as the truth for that question, and inconsistent responses were disregarded.
Gatekeeper	The response to the first question was taken as the truth, and all subsequent inconsistent responses were considered missing. If the response to question 1 (ever smoker) was no, regardless of the responses to subsequent questions, the current cigarette smoking status was assigned as noncurrent smoker. If the response to question 1 was yes, then current cigarette use status was defined by the response to question 3.
Global	Responses to all 6 questions were required to be consistent, and any inconsistent responses were considered missing.
Preponderance	Current cigarette smoking status, as defined by the answer to question 3, was assigned based on “preponderance of evidence” as determined by evaluation of responses. Responses to question 3 required consistency with responses on questions 4 through 6 regarding the past 30 days; otherwise, current cigarette use status was considered missing. Conversely, inconsistent or missing responses on current cigarette use status from question 3 could be reassigned if responses from questions 4 through 6 regarding the past 30 days were consistent.

We used Stata 11 software (StataCorp LP, College Station, Texas) to account for complex survey design and to calculate weighted point estimates and standard error (SE) of the estimates. Estimates with a relative SE (ratio of the SE of the estimate to the estimate, multiplied by 100) greater than 30% were considered statistically unreliable. Adjusted Wald tests were used to evaluate for statistical differences between point estimates derived from the GYTS approach and the 4 other data editing approaches. Significance was set at *P* < .05.

## Results

Overall response rates of students interviewed (calculated as the school response rate multiplied by the class and student response rates) for all 6 countries were the following: 84.0% (Ghana), 79.6% (Guatemala), 82.1% (Saudi Arabia), 80.9% (Philippines), 86.1% (Slovakia), and 93.1% (Thailand). Data edit approaches resulted in variation of prevalence estimates of cigarette use; estimates ranged from 2.3% to 5.1% in Ghana, 8.9% to 12.4% in Guatemala, 4.9% to 6.5% in Saudi Arabia, 12.3% to 17.0% in the Philippines, 21.6% to 25.0% in Slovakia, and 9.6% to 11.9% in Thailand (Table 2). The global approach resulted in lower estimates and the preponderance approach, in general, higher estimates. The do-nothing and gatekeeper approaches produced estimates similar to those of the GYTS approach. The range and magnitude of differences in estimates derived from the global and preponderance approaches compared with those of the GYTS approach were greater among girls than boys. All comparisons of GYTS estimates were significantly different (*P* < .05) from estimates derived with the 4 other approaches, with several exceptions (Table 2). Consistent with the overall estimates, the global approach resulted in lower estimates, the preponderance approach higher estimates, and the do-nothing and gatekeeper approaches similar estimates, by sex across all selected countries.

**Table 2:** Prevalence^a^ of Cigarette Use Among Global Youth Tobacco Survey (GYTS) Participants Aged 13–15 Years in Select Countries^b^, by Data Editing Approach

Country	Data Editing Approach									
	GYTS		Do-nothing		Gatekeeper		Global		Preponderance	
	n	% (SE)	n	% (SE)	n	% (SE)	n	% (SE)	n	% (SE)
**Ghana**										
Total	3,760	3.6 (0.8)	3,764	3.6 (0.8)	3,839	3.5 (0.8)	3,028	2.3 (0.5)	3,690	5.1 (1.0)
Boys	1,795	4.3 (1.0)	1,797	4.3 (1.0)	1,830	4.3 (0.9)	1,439	2.8 (0.5)	1,757	5.7 (1.1)
Girls	1,965	2.9 (0.8)	1,967	2.9 (0.8)	2,009	2.8 (0.8)	1,589	1.9 (0.7)^c^	1,933	4.4 (1.0)
**Guatemala**										
Total	3,433	11.3 (1.0)	3,468	11.2 (1.0)	3,518	11.1 (1.0)	3,020	8.9 (0.8)	3,352	12.4 (1.0)
Boys	1,536	13.8 (1.5)	1,553	13.7 (1.5)	1,570	13.5 (1.5)	1,351	11.3 (1.4)	1,501	15.4 (1.5)
Girls	1,897	9.1 (1.2)	1,915	9.1 (1.1)	1,948	8.9 (1.1)	1,669	6.7 (0.9)	1,851	9.7 (1.2)
**Saudi Arabia**										
Total	2,352	6.2 (0.8)	2,356	6.2 (0.8)	2,371	6.1 (0.8)	2,106	4.9 (0.7)	2,255	6.5 (0.8)
Boys	1,031	10.2 (1.3)	1,031	10.2 (1.3)	1,041	10.1 (1.3)	900	8.6 (1.2)	982	10.8 (1.3)
Girls	1,321	2.6 (0.9)^c^	1,325	2.6 (0.9)^c^	1,330	2.6 (0.9)^c^	1,206	1.9 (0.6)^c^	1,273	2.8 (1.0)^c^
**Philippines**										
Total	3,033	14.2 (1.4)	3,207	17.0 (1.5)	3,215	17.0 (1.5)	2,681	12.3 (1.2)	3,014	15.6 (1.4)
Boys	1,229	20.2 (2.0)	1,326	23.4 (2.0)	1,327	23.4 (2.0)	1,041	18.7 (1.9)	1,220	22.3 (2.1)
Girls	1,804	9.5 (1.3)	1,881	12.0 (1.4)	1,888	11.9 (1.4)	1,640	7.6 (1.3)	1,794	10.3 (1.4)
**Slovakia**										
Total	3,931	24.8 (1.2)	3,948	24.9 (1.2)	3,958	24.8 (1.2)	3,171	21.6 (1.4)	3,832	25.0 (1.3)
Boys	1,893	26.4 (1.6)	1,902	26.5 (1.6)	1,908	26.4 (1.6)	1,547	24.8 (1.9)	1,840	26.6 (1.7)
Girls	2,038	23.3 (1.4)	2,046	23.4 (1.4)	2,050	23.4 (1.4)	1,624	18.5 (1.4)	1,992	23.5 (1.4)
**Thailand**										
Total	7,368	11.6 (0.8)	7,368	11.6 (0.8)	7,392	11.6 (0.8)	6,675	9.6 (0.8)	7,217	11.9 (0.8)
Boys	3,075	20.1 (1.4)	3,075	20.1 (1.4)	3,085	20.0 (1.4)	2,682	17.4 (1.4)	3,012	20.8 (1.4)
Girls	4,293	3.8 (0.4)	4,293	3.8 (0.4)	4,307	3.8 (0.4)	3,993	3.0 (0.4)	4,205	3.8 (0.5)

Abbreviation: SE, standard error.

a Estimates are derived from a final sample of nonmissing data on sex and from questions 1 through 6 listed in Table 1; therefore, slight differences may exist when comparing data with those from country fact sheets.

b All comparisons of GYTS estimates were significantly different (*P* < .05) from estimates derived with the 4 other approaches with the following exceptions: there were no significant differences between the GYTS approach and the do-nothing approach for Ghana, Saudi Arabia, and Slovakia (both sex groups); between the GYTS approach and the gatekeeper and preponderance approaches for Slovakia (both sex groups); between the GYTS approach and the preponderance approach for Saudi Arabia (girls only); between the GYTS approach and the do-nothing approach (both sex groups); and between the GYTS approach and the preponderance approach (girls only) in Thailand.

c Estimates with relative SE higher than 30%; no estimates had a relative SE higher than 40%.

## Discussion

We demonstrated the effect of decision rules for handling data inconsistencies in GYTS data to assist collaborators globally. Smoking prevalence estimates generated from surveys can vary with the data editing approach used. Compared with the GYTS data edits, the global approach resulted in lower estimates and the preponderance approach, higher estimates. It is noteworthy that the do-nothing and gatekeeper approaches produced estimates similar to those of the GYTS data editing method. In comparison to the GYTS approach (7 logic checks), data editing methods in the National Youth Tobacco Survey and Youth Risk Behavior Survey are more extensive (more than 30 logic checks for each), suggesting a need to provide a more comprehensive list of logic checks to account for all possible combinations of inconsistencies in GYTS data (8,9).

This study shows how different ways of removing inconsistent data influence the degree to which cigarette smoking is estimated. Clearly described methods for handling inconsistent data are necessary for reproducibility and comparability of GYTS results. Multiple researchers across WHO regions use and publish GYTS data, and accurate comparisons between 2 studies can be made only if the same approach in handling inconsistent data is used. Resolving issues with data inconsistency may include piloting surveys before implementation and incorporating built-in skip patterns if electronic versions of the survey are explored in the future. A limitation of this study is that the list of sampled countries is not representative of, and therefore not generalizable to, all countries conducting GYTS.

Data cleaning and management, as essential aspects of quality assurance and determinants of study validity, require transparency and proper documentation of all procedures (10). Implications for researchers using GYTS include recognition of its data editing approach and documentation in their study methods to ensure cross-study comparability.
